# Supplementary material for: Characteristics and risk factors of infections in patients with HBV-related acute-on-chronic liver failure: a retrospective study
Source: PeerJ. 2022 Jul 5;10:e13519. doi: 10.7717/peerj.13519 (PMC9266584; doi:10.7717/peerj.13519)
Supplement: Supplemental Information 3 [file peerj-10-13519-s003.docx]

education

0=illiteracy; 1=primary school; 2=junior school; 3=senior school; 4=college; 5=postgraduate

quantity of diuretic

0=no use of diuretic; 1=one diuretic; and so on

frequency of diuretic

0=no use of diuretic; 1=use diuretics once a day; 2=use diuretics twice a day; and so on

other infections

0=no other site of infection; 1=complicated with one other site of infection; and so on

hepatic encephalopathy

0=no HE; 1=complicated with HE

hepatorenal syndrome

0=no HS; 1=complicated with HS

cirrhosis

0=no cirrhosis; 1=complicated with cirrhosis

stay in bed

0= no stay in bed; 1=stay in bed

quantity of antibiotic

0=no use of antibiotic; 1=one antibiotic; and so on

time of antibiotic

0=no use of antibiotic; 1=1-10 days; 2=11-20 days; 3=21-30 days; 4=≥ 31 days

HbeAg

0=negative for HbeAg; 1=positive for HbeAg

HBV DNA

0=negative; 1=low level copies; 2=high level copies; the specific instructions can be seen in methods of manuscript
